# Centroparietal periodic sharp-wave discharges and biphasic complexes: Novel EEG biomarkers for early diagnosis of Rasmussen encephalitis

**DOI:** 10.1016/j.ebr.2026.100873

**Published:** 2026-05-19

**Authors:** Philippe Gélisse, Pierre Genton, Arielle Crespel

**Affiliations:** a Epilepsy Unit, Gui de Chauliac Hospital, Montpellier, France; b Research Unit (URCMA: Unité de Recherche sur les Comportements et Mouvements Anormaux), INSERM, U661, Montpellier, France; c Neurology Department, Hôpital Saint Charles, 13100 Aix en Provence, France

**Keywords:** Electroencephalogram, Focal seizures, Drug-resistance, Rasmussen syndrome, Immunotherapy, Adalimumab

## Abstract

**Background:**

Rasmussen encephalitis (RE) is a rare, chronic inflammatory brain disorder that predominantly affects one hemisphere in children. It is characterized by progressive neurological deterioration and refractory seizures. Early diagnosis remains challenging due to nonspecific initial symptoms and diagnostic features that overlap with other neurological conditions.

**Objective:**

To identify early and specific EEG patterns that may support timely initiation of immunomodulatory therapies.

**Method:**

Continuous long-term video-EEG monitoring was repeatedly conducted in a female patient who experienced her first seizures at age 16, soon after a febrile illness.

**Results:**

Identification of two distinctive EEG patterns—focal periodic sharp-wave discharges and biphasic complexes—allowed early diagnosis prior to the onset of severe deficits or epilepsia partialis continua. The biphasic complexes occurred alongside unilateral slow-wave activity, whereas focal periodic sharp-wave discharges were initially observed on an otherwise normal background. The biphasic complexes appear to herald evolution to the acute phase of the disease. Prompt immunomodulatory therapy (intravenous immunoglobulin followed by adalimumab) successfully halted disease progression, nearly normalized EEG background activity, and reduced seizure frequency.

**Conclusions:**

This case underscores the prognostic value of recurrent focal periodic sharp-wave discharges and biphasic complexes as prodromal EEG biomarkers for RE, aligning with one prior study linking these biphasic complexes to early-stage disease (Beaumanoir et al., 1997). Early recognition of these patterns is essential. Early initiation of immunotherapy may prevent irreversible neurological damage, highlighting the critical role of recognizing these electrographic patterns for timely diagnosis and intervention.

## Highlights

• Rasmussen encephalitis (RE) is often diagnosed during the acute phase.
• EEG findings are crucial when an MRI appears normal.
• EEG findings, such as focal periodic sharp wave discharges and biphasic complexes, may flag early RE.
• EEG patterns differentiate RE from focal cortical dysplasia and other focal epilepsies.
• Early recognition enables prompt immunotherapy, which may help slow disease progression.

## 1 Introduction

Rasmussen encephalitis (RE) is a rare chronic inflammatory brain disease that affects one hemisphere and typically occurs in children, with a mean onset age of five years [1]. The disease progresses through three stages—a prodromal stage, followed by an acute stage characterized by frequent seizures, with epilepsia partialis continua in more than half of patients, progressive neurological deficits, and then a residual stage. According to the 2005 European Consensus, diagnosing RE relies on converging clinical, EEG, MRI, and histopathological evidence [2]. On EEG, the hallmark is slowing confined to one hemisphere, with or without epileptiform activity, and unilateral seizure onset. Early diagnosis of RE is difficult due to nonspecific initial symptoms and EEG and MRI findings that overlap with other neurological conditions. In the early stages, EEGs may appear near-normal with up to 20% of children showing normal results at 3 months and 12% at 6 months [3]. As the disease progresses, focal abnormalities develop, followed by hemispheric changes and characteristic focal ictal discharges [1], [4]. In most cases, a definitive diagnosis is reached in the acute phase, when the clinical picture—together with EEG and MRI features—becomes distinctly compatible with RE.

Beaumanoir et al. reported the detection of biphasic complexes during the prodromal phase of RE, preceding clinical evidence of chronic encephalitis [5]. We report an adolescent with a distinctive EEG phenotype, initially characterized by vertex–transient–like patterns and later displaying typical Beaumanoir's biphasic complexes concomitant with unilateral slowing. These electrographic features enabled an early diagnosis of RE—prior to the emergence of severe neurological deficits or epilepsia partialis continua—permitting the prompt initiation of immunomodulatory therapy. This intervention successfully halted disease progression, led to the near resolution of unilateral slowing on EEGs, and resolved a distal motor deficit.

## 2 Observation

**Fig. 1:**
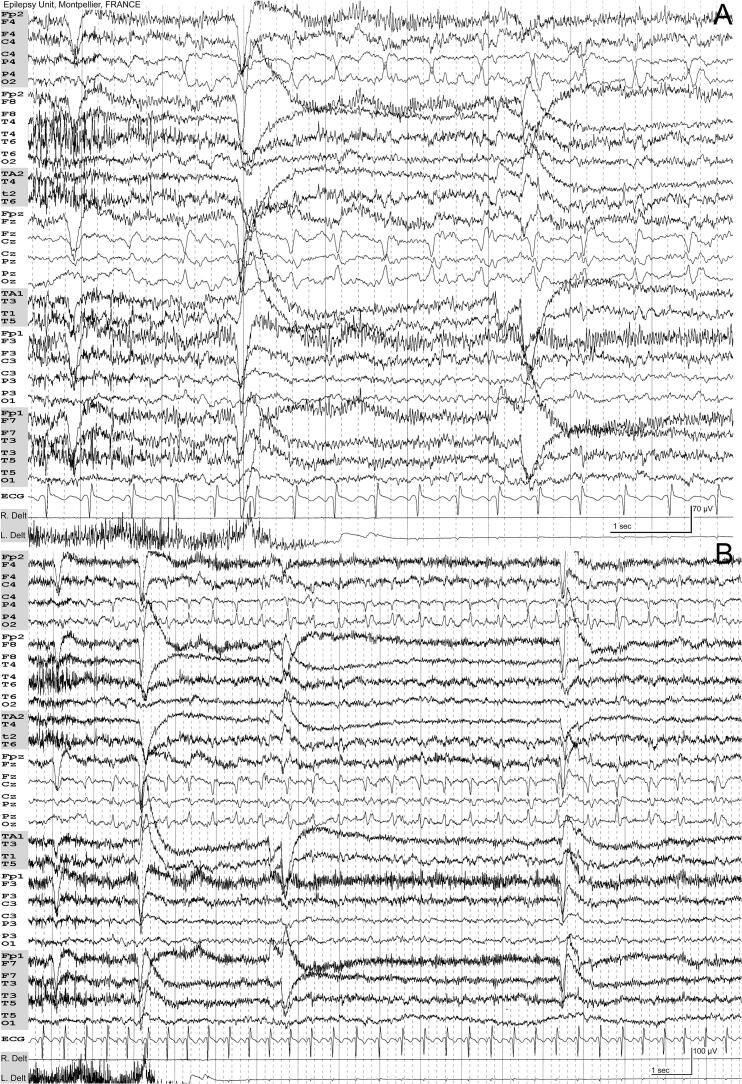
EEG recorded at 16.5 years of age. International 10–20 electrode placement system and supplementary anterior/inferior temporal electrodes (TA1/TA2: Temporal-Anterior; T1/T2: zygomatic electrode). Plate A (30 mm/s, Awake): A focal subclinical seizure characterized by periodic sharp waves localized on the vertex and right parietal region. Plate B (15 mm/s): The same EEG segment displayed at a reduced paper speed to show the full duration of the discharge.

A 16-year-old girl presented with stereotyped focal seizures characterized by a distinct sensory aura which originated in the left foot and progressively ascended the leg. These episodes frequently evolved into involuntary tonic contractions of the left foot and, less often, the left arm. Her first seizure occurred shortly after a febrile illness with rhinitis and a cough. Despite treatment with oxcarbazepine and clobazam, the patient continued to experience persistent seizures, with a maximum seizure-free interval of seven days. Six months after symptom onset, she underwent 48-h video-EEG monitoring for further evaluation. Background activity was normal, and NREM sleep demonstrated symmetrical, well-preserved physiological sleep elements. The most notable finding was the presence of sharp wave discharges, localized on the vertex and the right centroparietal regions mimicking vertex waves. These epileptiform discharges occurred in isolated or brief bursts during both wakefulness and NREM sleep (Fig. 1; Supplementary), but without sleep activation. Occasionally, they formed short periodic runs lasting 10–15 s, during which the patient (if awake) could exhibit a transient loss of muscle strength in the left foot—but most of the time, these discharges were subclinical.

Six months later, a follow-up EEG again showed vertex-transient-like discharges with normal symmetry in both background activity and NREM sleep elements. During hyperventilation, however, there was right hemispheric slow wave activity not previously observed. Brain MRI, adjusted to population norms, demonstrated mild atrophy of the right parietal region. PET scan imaging revealed hypometabolism in the right parietal lobe extending into the right temporo insular region (Supplementary). The initial CSF analysis was unremarkable.

**Fig. 2:**
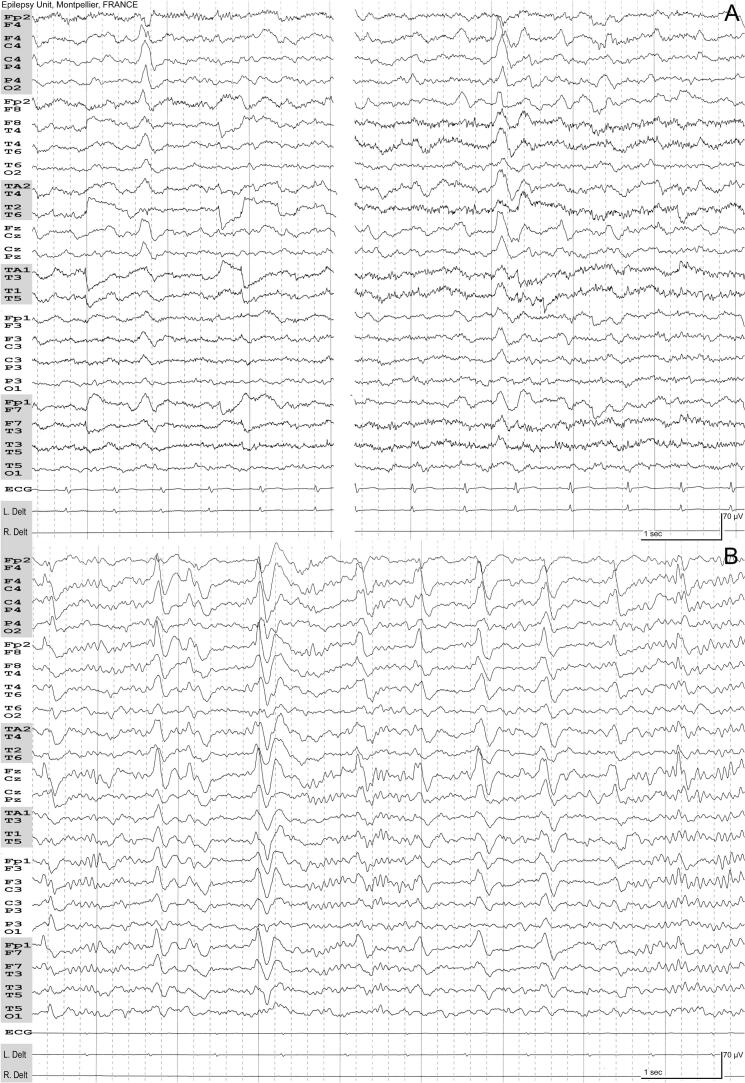
EEG recorded at 17.5 years of age. International 10–20 electrode placement system and supplementary anterior/inferior temporal electrodes (TA1/TA2: Temporal-Anterior; T1/T2: zygomatic electrode). Plate A (Awake): Isolated biphasic complexes are observed on the fronto-central and vertex regions. Note the slowing of background activity on the right hemisphere. Plate B (NREM Sleep): A series of biphasic complexes is present. Sleep spindles are more prominent on the left hemisphere.

A follow-up EEG six months later revealed slowing of background activity in the right hemisphere, with frequent subclinical focal seizures—most prominent during nocturnal sleep—characterized by periodic or rhythmic activity on the right frontocentral or the vertex regions. Additional focal seizures featured tonic contraction of the left foot. Unlike the initial EEGs, typical frontocentral Beaumanoir's biphasic complexes were now present in wakefulness, NREM sleep, and REM sleep (Fig. 2; Supplementary). During this period, the patient developed persistent weakness of the left foot and began experiencing atypical seizures described as a sensation of bodily fragmentation—she referred to these as “Guernica”—which could recur several times per day. She also had a left hemiclonic seizure approximately every two weeks. Neuropsychological evaluation remained unremarkable, with no detectable deficits. A second CSF examination demonstrated three distinct oligoclonal bands, consistent with intrathecal immunoglobulin synthesis.

Monthly infusions of polyvalent intravenous immunoglobulin were initiated in conjunction with levetiracetam, carbamazepine, lacosamide, and clonazepam, and continued for two years. This regimen produced a clear clinical benefit, lowering the frequency of hemiclonic seizures and reducing the patient's characteristic “Guernica” seizures to approximately one episode per month. The brief seizures that did occur were typically limited to a sensory aura in the left foot or transient focal weakness. A 48-h video-EEG indicated persistent right-hemispheric background slowing, ongoing biphasic complexes, and focal subclinical seizures—although these were less frequent than on the EEG two years prior. A neurological examination showed normal strength, with no evidence of weakness.

Polyvalent immunoglobulins were discontinued and replaced with biweekly adalimumab, leading to a marked EEG improvement which included near normalization of background activity and the reemergence of physiological sleep patterns on the right hemisphere. The patient achieved prolonged seizure-free intervals lasting several days. After 14 months of treatment, adalimumab was withdrawn. Notably, seizure frequency remained stable at the one-year follow-up. The patient reported only focal seizures with transient “blocking” of left foot movement, and no “Guernica” seizures.

## 3 Discussion

There is significant interest in early diagnosis; identifying the first signs of RE could enable the timely initiation of immunomodulatory therapies, an early intervention that may slow disease progression and prevent severe neurological deterioration. Early EEG recordings rarely distinguish RE from other focal epilepsies [4], such as focal cortical dysplasia [3]. RE is often diagnosed in the setting of refractory focal motor seizures. The combination of this seizure phenotype with contralateral EEG slowing and specific neuroimaging changes can support a diagnosis of RE as early as 4–6 months after symptom onset [6]. The pattern of ictal periodic discharges that we observed—most of which were subclinical—is highly unusual in focal epilepsy. Indeed, in focal cortical dysplasia, the interictal EEG typically shows focal spikes or spike-and-wave complexes, fast epileptic rhythms localize to the dysplastic cortex, and seizures are characterized by an evolving rhythmic pattern that increases in amplitude and progressively involves adjacent regions [7].

One study has specifically focused on the early electroencephalographic features of RE during the prodromal phase. Beaumanoir et al., described biphasic complexes in two children aged 4.5 and 3 years, respectively. Both patients presented with a febrile syndrome preceding their initial seizures. These stereotyped complexes were observed on EEG immediately after the first seizures. Present in both waking and sleep EEG recordings, they were associated with polymorphic slow-wave activity, and later with multifocal spikes. Ultimately, both children developed a typical form of RE, characterized by epilepsia partialis continua and hemiparesis. According to the authors, these complexes suggested a chronic focal encephalitis as the underlying cause of the focal epilepsy—even though the presentation of epilepsy did not appear severe, and no motor or intellectual deficits were initially anticipated. Notably, the detection of focal biphasic complexes in children—particularly when observed interictally (i.e., distant from seizure activity) and in the context of a benign-appearing febrile illness—should prompt consideration of RE as a potential diagnosis.

The morphology of the biphasic complexes in our patient's right fronto-central region closely corresponds to those described by Beaumanoir et al. These complexes were also observed in the context of hemispheric slowing and in a patient who, 18 months earlier, had a febrile infection before the first seizure. However, in contrast to Beaumanoir et al., these complexes were accompanied by frequent daily focal seizures (both clinical and subclinical, particularly during nocturnal sleep). Vertex-transient-like patterns, even when occurring as single elements, did not exactly match the morphology of the biphasic complexes described by Beaumanoir et al., despite appearing biphasic on average montage (Supplementary). This discrepancy may reflect their differing locations—near the midline in our recordings versus over the convexity in the cases of Beaumanoir et al. Although our patient did not exhibit epilepsia partialis continua, motor deficits, or clear changes on MRI, the presence of these EEG findings—along with unilateral slowing; recurrent clinical and subclinical seizures; and marked hypometabolism on PET—strongly supported a diagnosis of RE.

In conclusion, recurrent focal periodic sharp-wave discharges and Beaumanoir's biphasic complexes appear to be distinctive EEG hallmarks of RE during the prodromal phase, preceding epilepsia partialis continua or significant motor deficits. Early recognition of these patterns is essential—it enables timely initiation of immunotherapy in appropriate cases and may mitigate disease progression. Typical Beaumanoir's biphasic complexes occurred alongside unilateral slow-wave activity whereas vertex-transient-like patterns and focal periodic sharp-wave discharges were initially observed on an otherwise normal background. The typical Beaumanoir's biphasic complexes seem to herald evolution to the acute phase. In a child or an adolescent with focal motor seizures, their presence—together with unilateral background slowing—should prompt consideration of RE, even when the brain MRI is unrevealing or shows no significant change.

## CRediT authorship contribution statement

**Philippe Gélisse:** Conceptualization, Methodology, Supervision, Validation, Writing – original draft, Writing – review & editing. **Pierre Genton:** Writing – review & editing. **Arielle Crespel:** Writing – review & editing.

## Ethical statement

We confirm that we have read the Journal's position on issues involved in ethical publication and affirm that this report is consistent with those guidelines.

The authors certify that no work resembling the enclosed article has been published or is being submitted elsewhere. We also certify that we have made a substantial contribution so as to qualify for authorship. We have disclosed all financial support for our work and other potential conflicts of interest.

## Declaration of competing interest

Dr. Gélisse received support for teaching programs from UCB, Eisai, Angelini, honoraria from Livanova, and royalties for publishing from John Libbey Eurotext.

Dr. Genton received royalties for publishing from John Libbey Eurotext.

Dr. Crespel received support for teaching programs from UCB, Eisai, Angelini; honoraria from Eisai, Angelini, and royalties for publishing from John Libbey Eurotext.
