# Supplementary material for: Centroparietal periodic sharp-wave discharges and biphasic complexes: Novel EEG biomarkers for early diagnosis of Rasmussen encephalitis
Source: Epilepsy Behav Rep. 2026 May 19;34:100873. doi: 10.1016/j.ebr.2026.100873 (PMC13223952; doi:10.1016/j.ebr.2026.100873)
Supplement: Supplementary file 1 — Supplementary material [file mmc1.pdf]

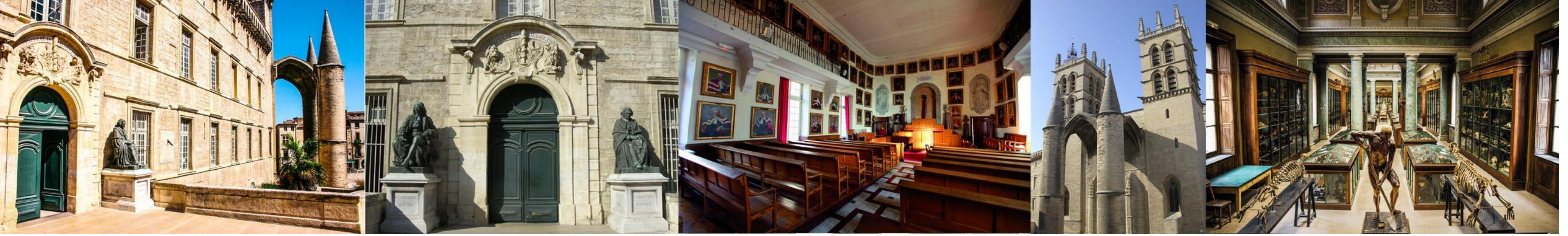

The Faculty of Medicine of Montpellier, created in the 12th century, is the oldest in the western world still in operation

Centroparietal periodic sharp-wave discharges and biphasic complexes: Novel EEG biomarkers for early diagnosis of Rasmussen encephalitis

Philippe GELISSE[1], Pierre GENTON[2], Arielle CRESPEL[1]

[1]Epilepsy Unit, Montpellier, France

[2]Neurology department, Aix-en-Provence, FRANCE

Highlights

- Rasmussen encephalitis (RE) is often diagnosed during the acute phase.
- EEG findings—such as focal periodic sharp wave discharges and biphasic complexes—may flag early RE.
- Early recognition enables prompt immunotherapy, which may help slow disease progression.

Anomalies EEG dans la phase prémonitoire du syndrome de Rasmussen. À propos de deux observations

A Beaumanoir [1], D Grioni [2], G Kullmann [2], A Tiberti [3], D Valseriati [3]

[1] *Fondazione P e L Mariani, Viale Bianca Maria 28, Milano ;* [2] *Clinica NP infantile, Ospedale generale, Monza ;* [3] *Divisione de NP infantile, Ospedale Brescia, Brescia, Italy*

Summary – Electroencephalographic abnormalities in the prodromic phase of the Rasmussen's syndrome. Study of two cases. *Electroencephalographic (EEG) recordings were studied at disease onset in two subjects presenting with Rasmussen's syndrome. Particular attention was paid to abnormalities detected during the prodromic phase before clinical outcome suggested the existence of chronic encephalitis. EEG recordings showed focal, polymorphic abnormalities associated with slow biphasic complexes (SBC). These complexes that are composed of two slow waves with opposite polarity, a 150- to 250-mV peak-to-peak amplitude and a 500-ms duration have only been described in inflammatory syndromes of the central nervous system. Their occurrence at onset of Rasmussen's syndrome are discussed.*

Beaumanoir A, Grioni D, Kullmann G, Tiberti A, Valseriati D. Anomalies EEG dans la phase prémonitoire du syndrome de Rasmussen. A propos de deux observations [EEG anomalies in the prodromic phase of Rasmussen's syndrome. Report of two cases]. Neurophysiol Clin. 1997;27:25-32. Doi: 10.1016/s0987-7053(97)89867-x..

Reproduced with permission

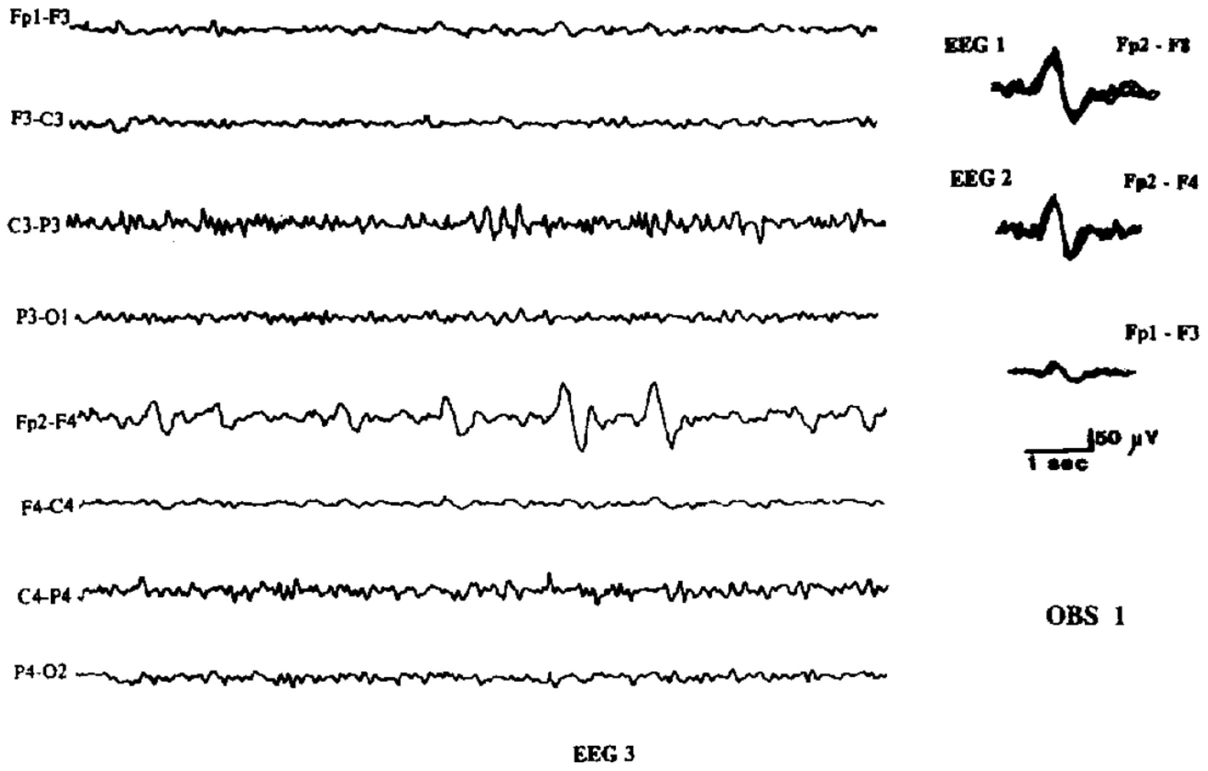

Fig 1. Cas 1. Enregistrement électroencéphalographique (EEG 3) des hémisphères gauche (dérivations Fp1-F3, F3-C3, C3-P3, P3-O1) et droit (dérivations Fp2-F4, F4-C4, C4-P4, P4-O2) et superposition de cinq complexes lents biphasiques (CLB). Enregistrement des CLB effectués 48 h après la première crise dans la région frontotemporale droite (EEG 1) et 11 jours après la première crise avec des CLB dans la région frontotemporale droite, ainsi que 5 jours après la deuxième crise avec des CLB dans les deux régions frontotemporales (EEG 2).

Fig 1. Case 1. Electroencephalographic recording (EEG 3) of the left hemisphere (derivations Fp1-F3, F3-C3, C3-P3, P3-O1) and right hemisphere (derivations Fp2-F4, F4-C4, C4-P4, P4-O2) with the superimposition of five slow biphasic complexes (SBC). Recording of SBC performed 48 hours after the first seizure in the right frontotemporal region (EEG 1) and 11 days after the first seizure with SBC in the right frontotemporal region as well as 5 days after the second seizure with SBC in both frontotemporal regions (EEG 2).

Reproduced with permission

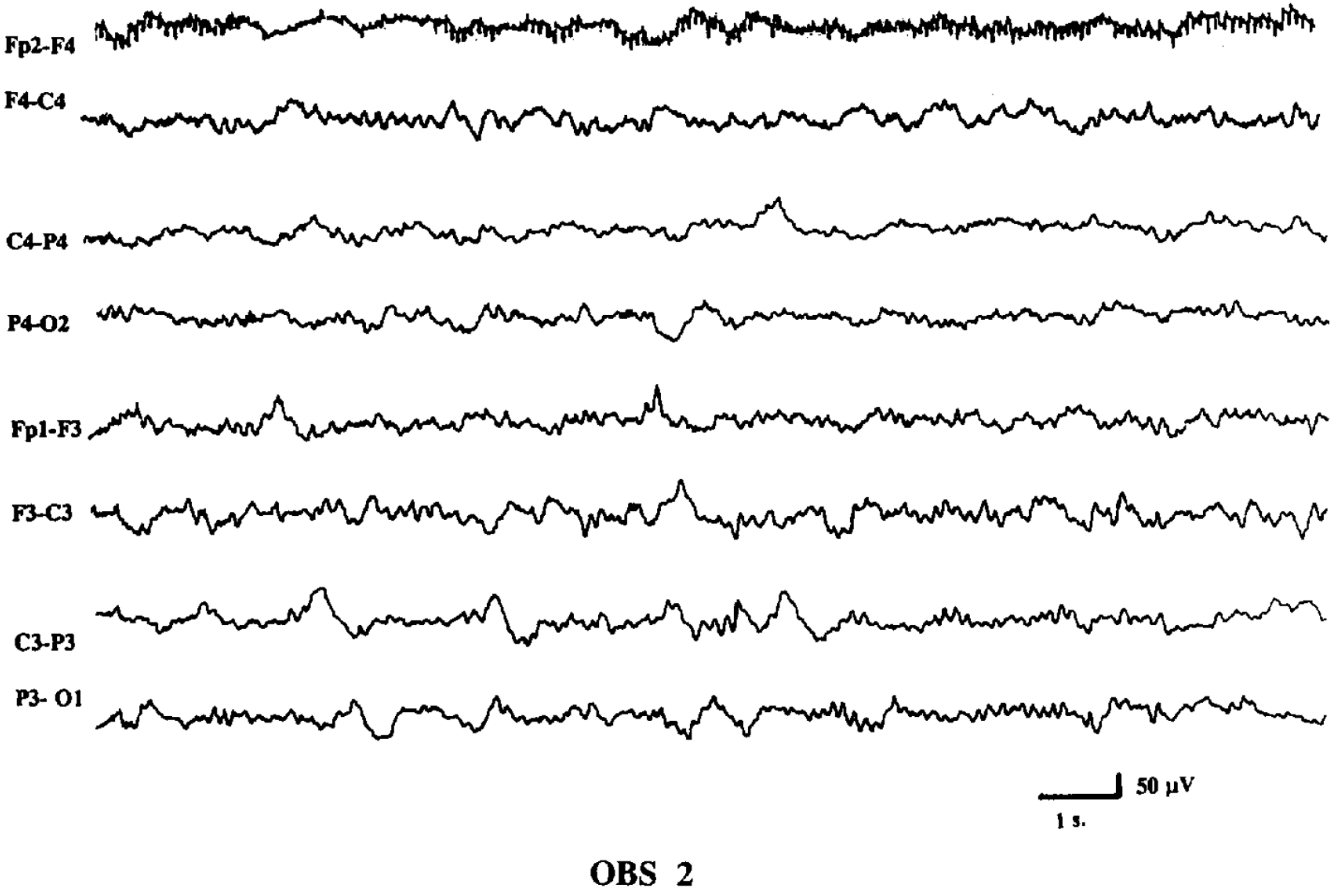

Fig 2. Cas 2. CLB dans la région postérieure gauche.

Fig 2. Case 2. Slow biphasic complexes (SBC) in the posterior left region.

Reproduced with permission

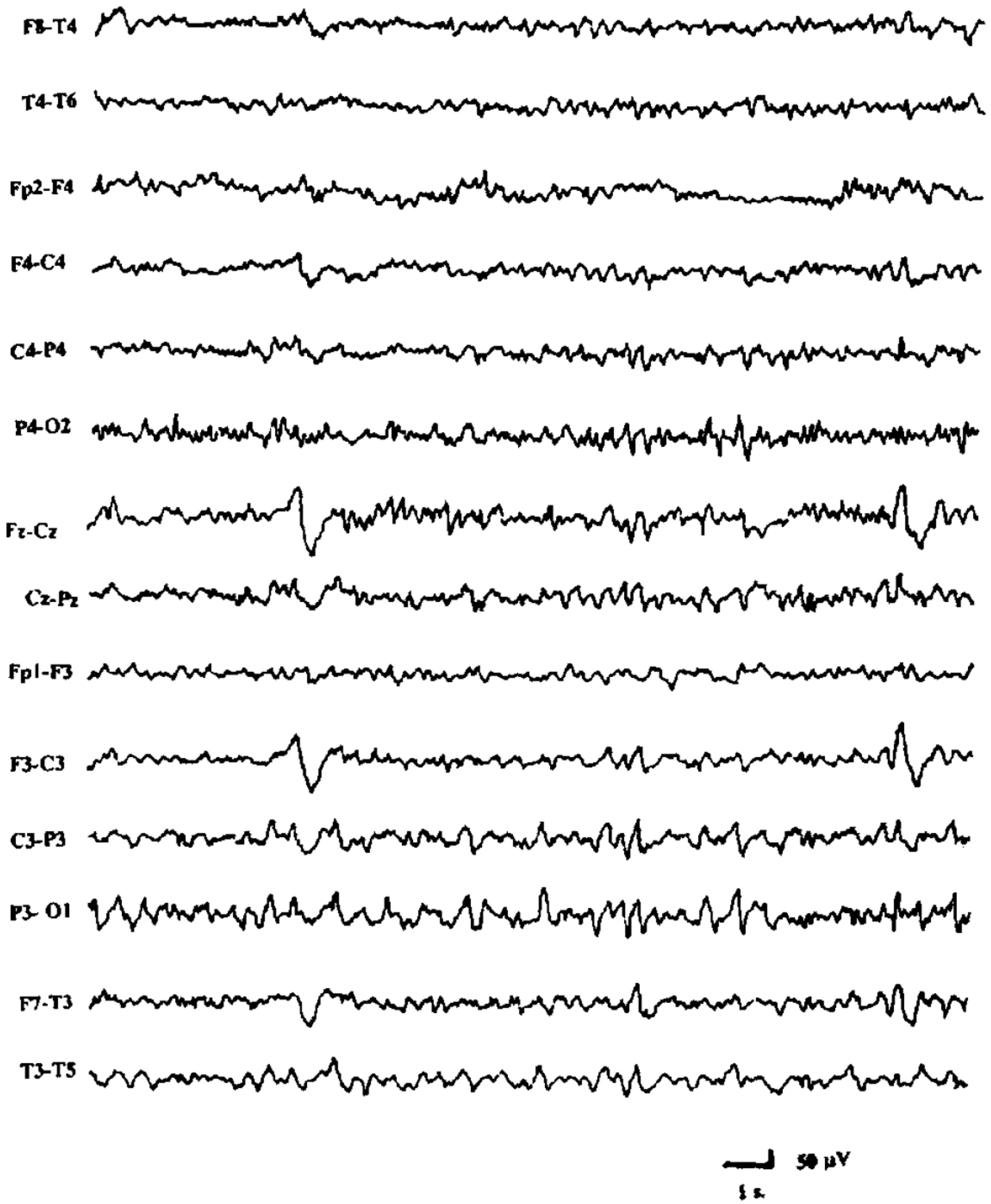

OBS 2

Fig 3. *Case 2. Recording at 4 months of progression, one week after an episode of asthenia with conjunctivitis: left parieto-occipital spikes; anterior left slow biphasic complexes (SBC).*

Fig 3. Cas 2. Enregistrement au 4e mois d'évolution, une semaine après un épisode d'asténie avec conjonctivite : pointes pariéto-occipitales gauches; CLB antérieures gauches.

EEG recorded at 16.5 years of age.

Awake. Focal subclinical seizure with periodic sharp waves occurring on the vertex and right parietal region.

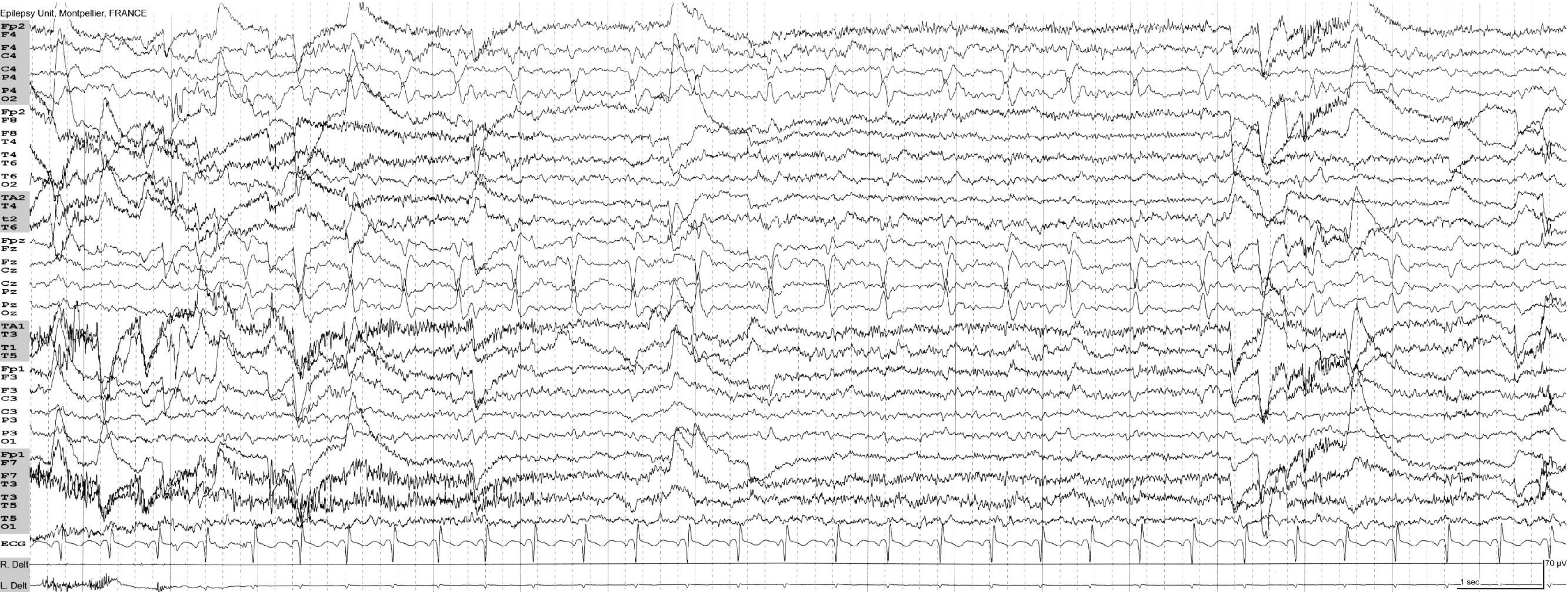

International 10–20 electrode placement system and supplementary anterior/inferior temporal electrodes

(TA1/TA2: Temporal-Anterior; T1/T2: zygomatic electrode)

EEG recorded at 16.5 years of age.

Awake. Focal subclinical seizure with periodic sharp waves occurring on the vertex and right parietal region.

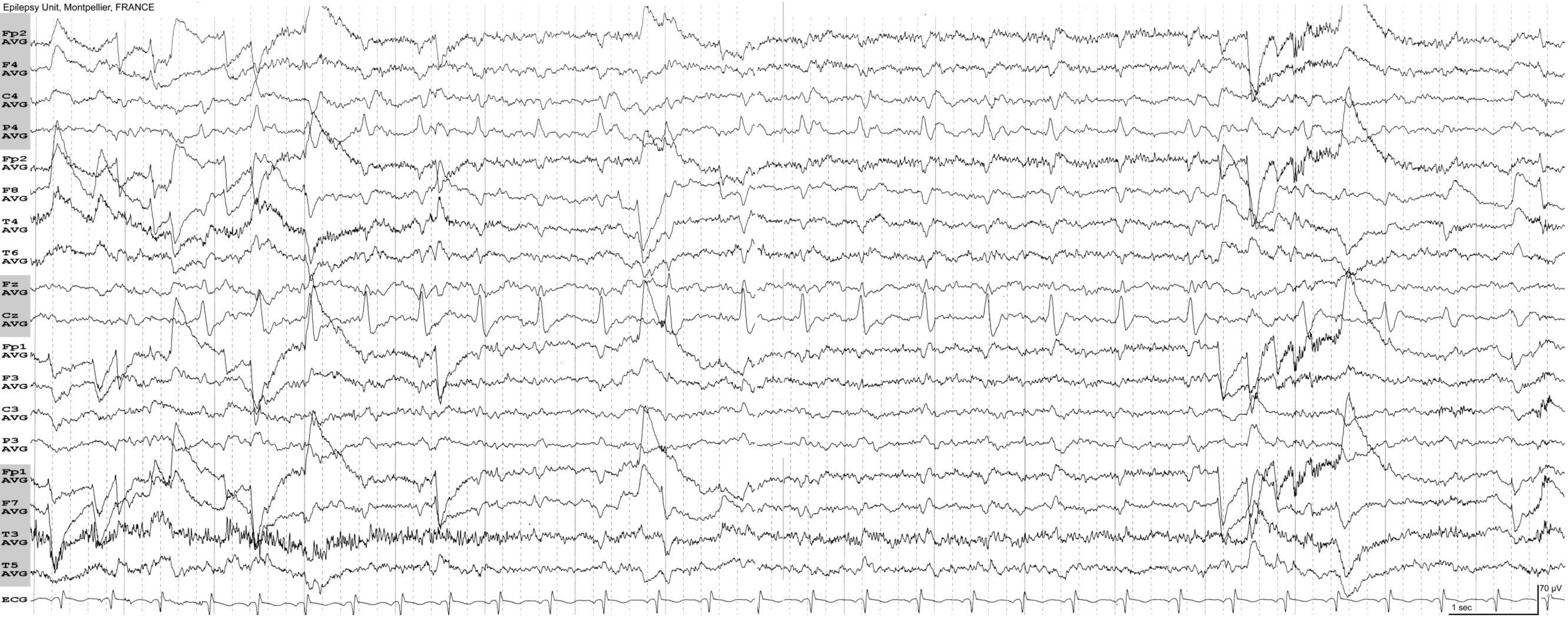

Same EEG panel. Average montage

EEG recorded at 16.5 years of age.

Awake. Focal subclinical seizure with periodic sharp waves occurring on the vertex and right parietal region.

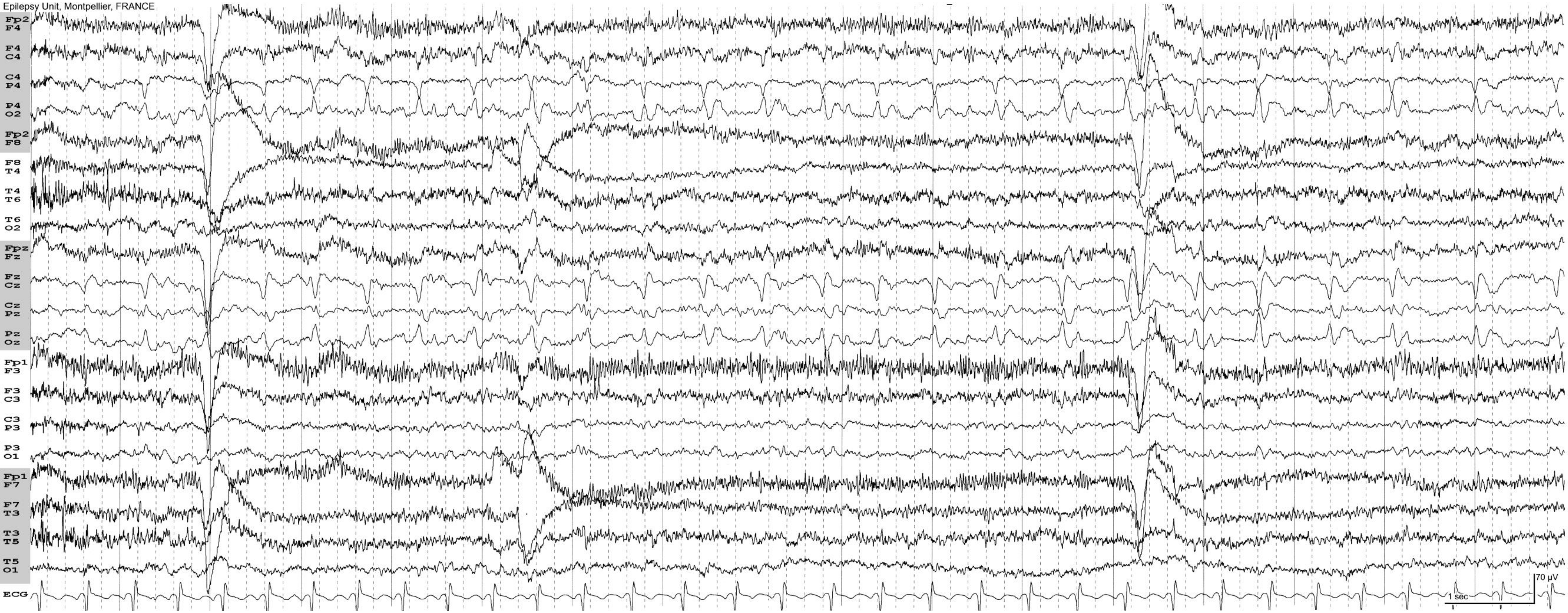

International 10–20 electrode placement system and supplementary anterior/inferior temporal electrodes (TA1/TA2: Temporal-Anterior; T1/T2: zygomatic electrode)

17-year-old

PET scan

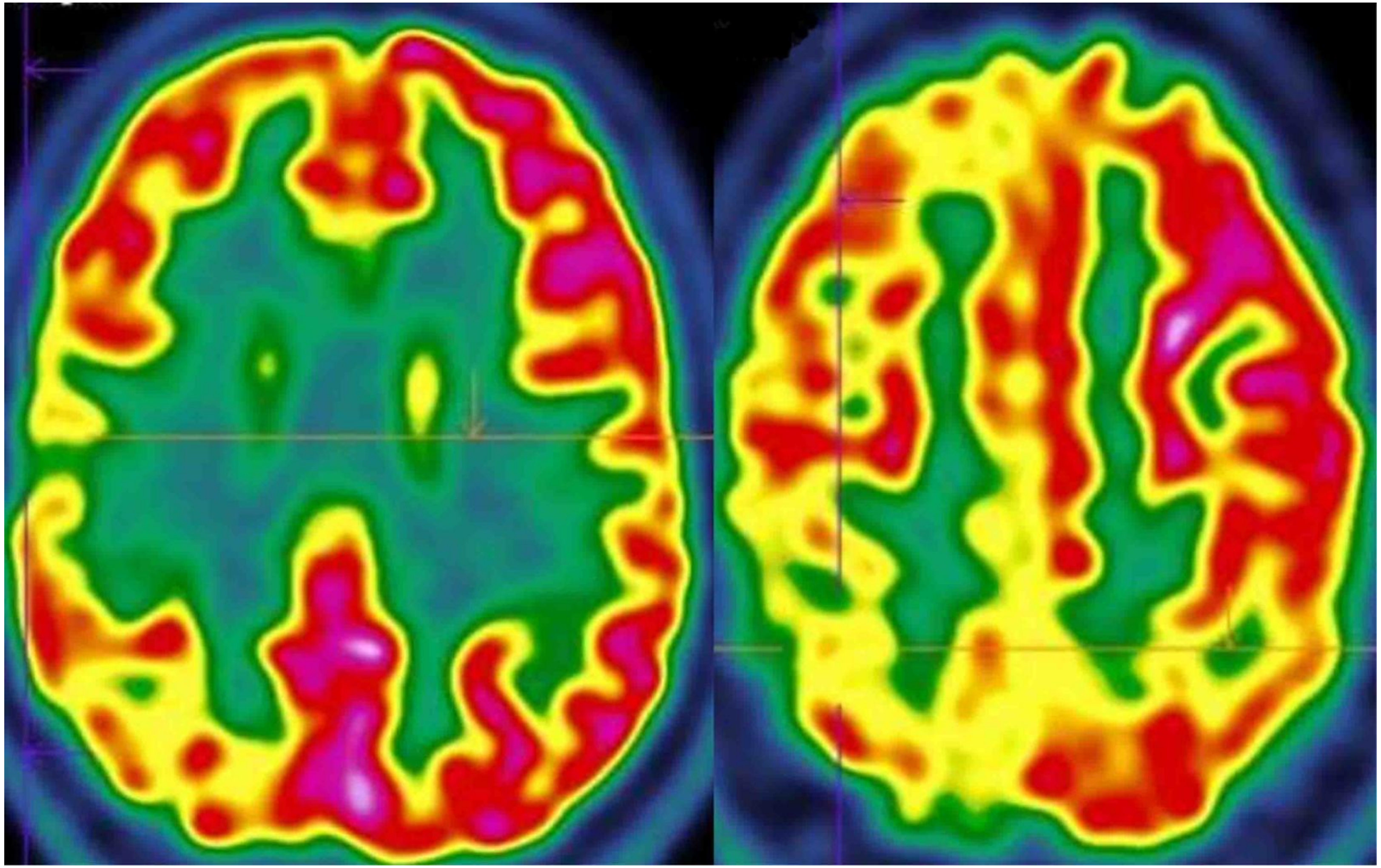

Hyypometabolism in the right parietal lobe.

EEG recorded at 16.5 years of age.

Awake. Focal subclinical seizure with periodic sharp waves occurring on the vertex and right parietal region.

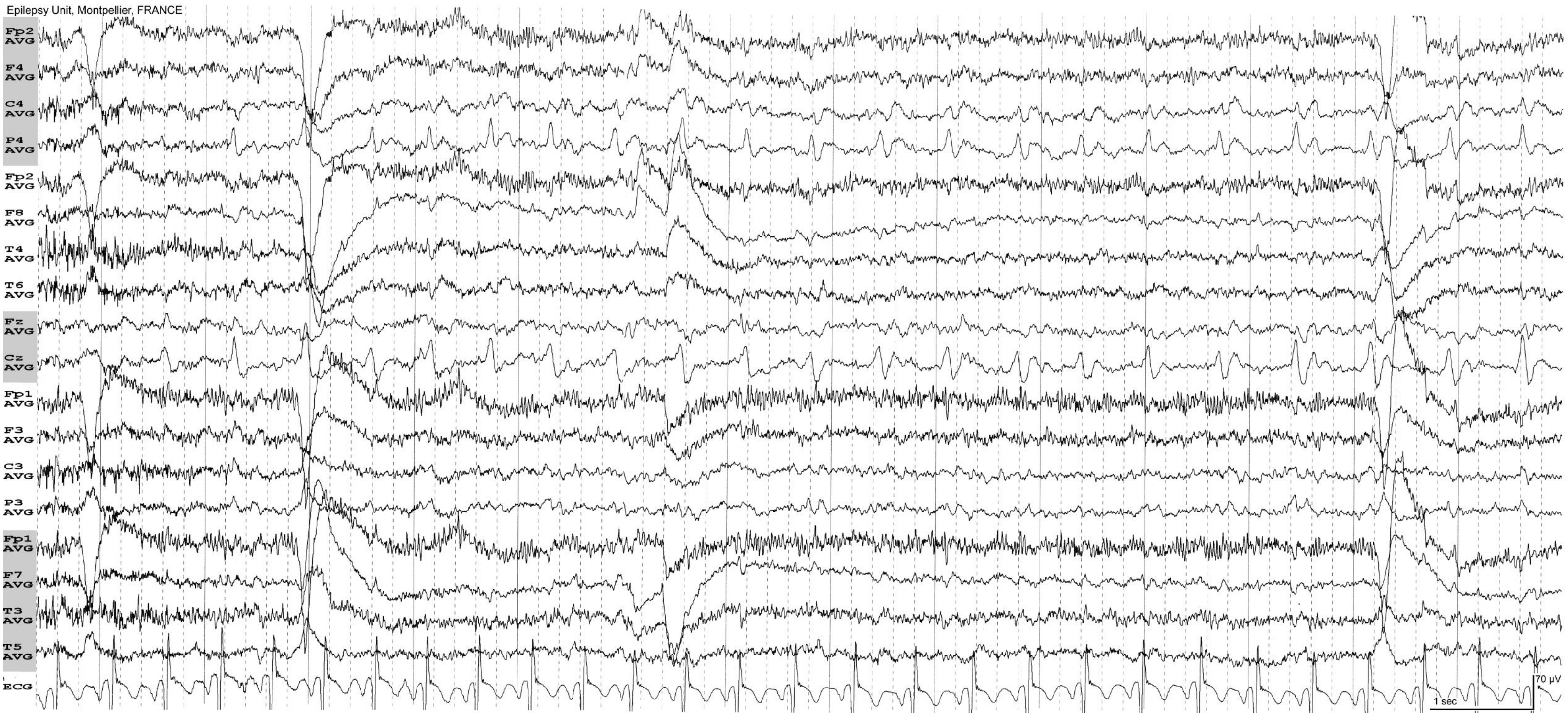

Same EEG panel. Average montage

EEG recorded at 16.5 years of age.

NREM sleep stage 1-2: vertex-transient-like patterns

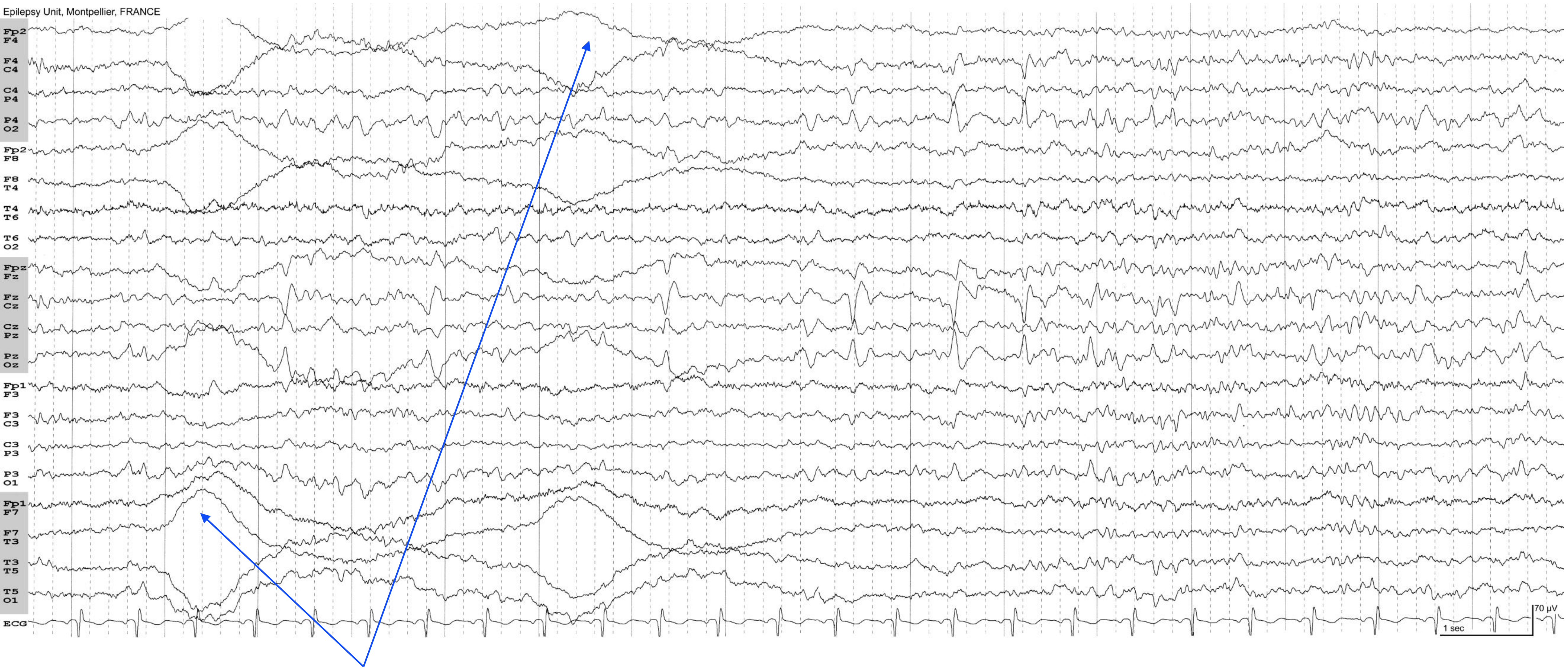

Sweat artifact

EEG recorded at 17.5 years of age.

Awake. Isolated biphasic complexes are observed on the fronto-central and vertex regions. Note the slowing of background activity on the right hemisphere.

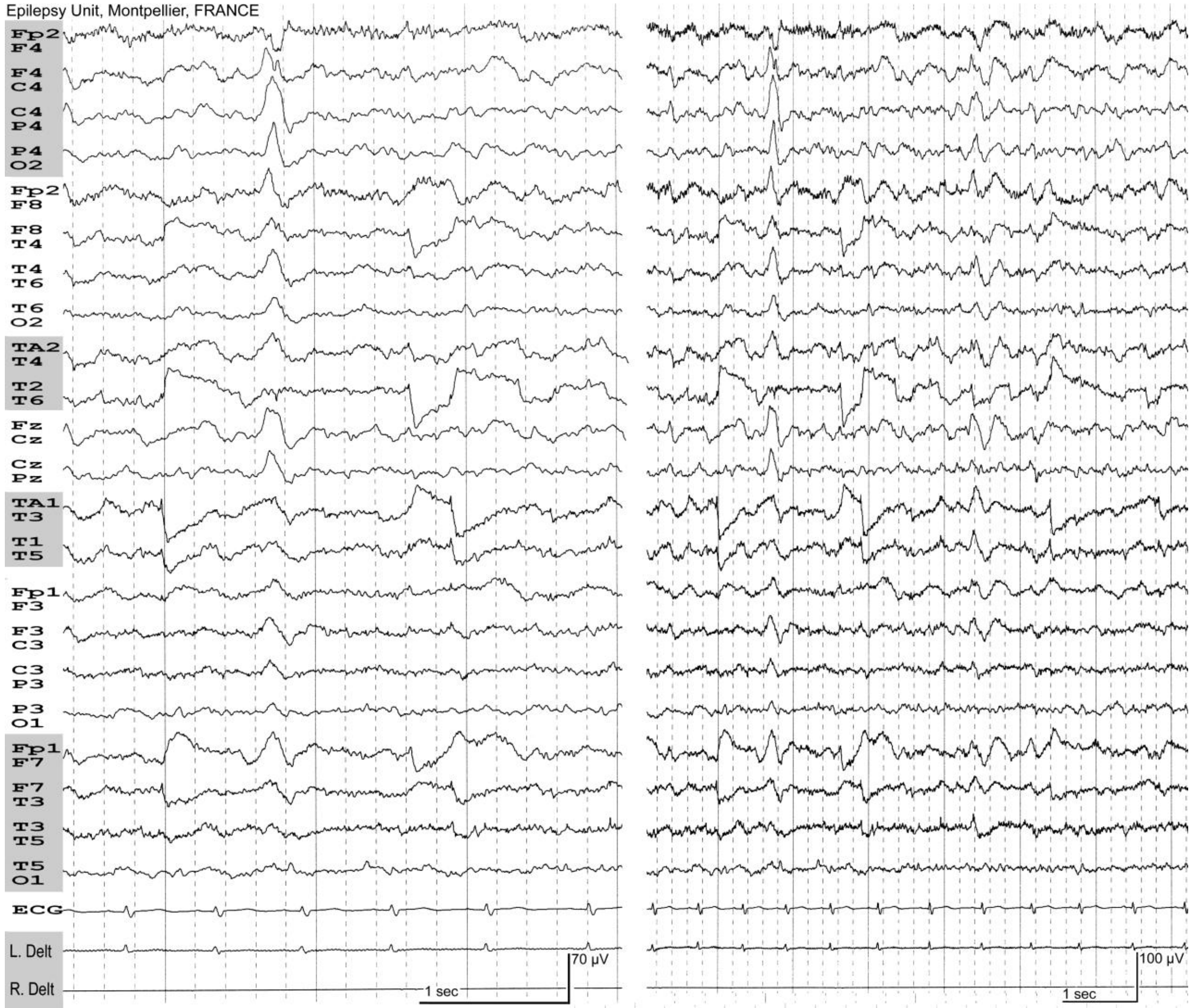

International 10–20 electrode placement system and supplementary anterior/inferior temporal electrodes (TA1/TA2: Temporal-Anterior; T1/T2: zygomatic electrode)

EEG recorded at 17.5 years of age.

Awake. Isolated biphasic complexes are observed on the fronto-central and vertex regions. Note the slowing of background activity on the right hemisphere.

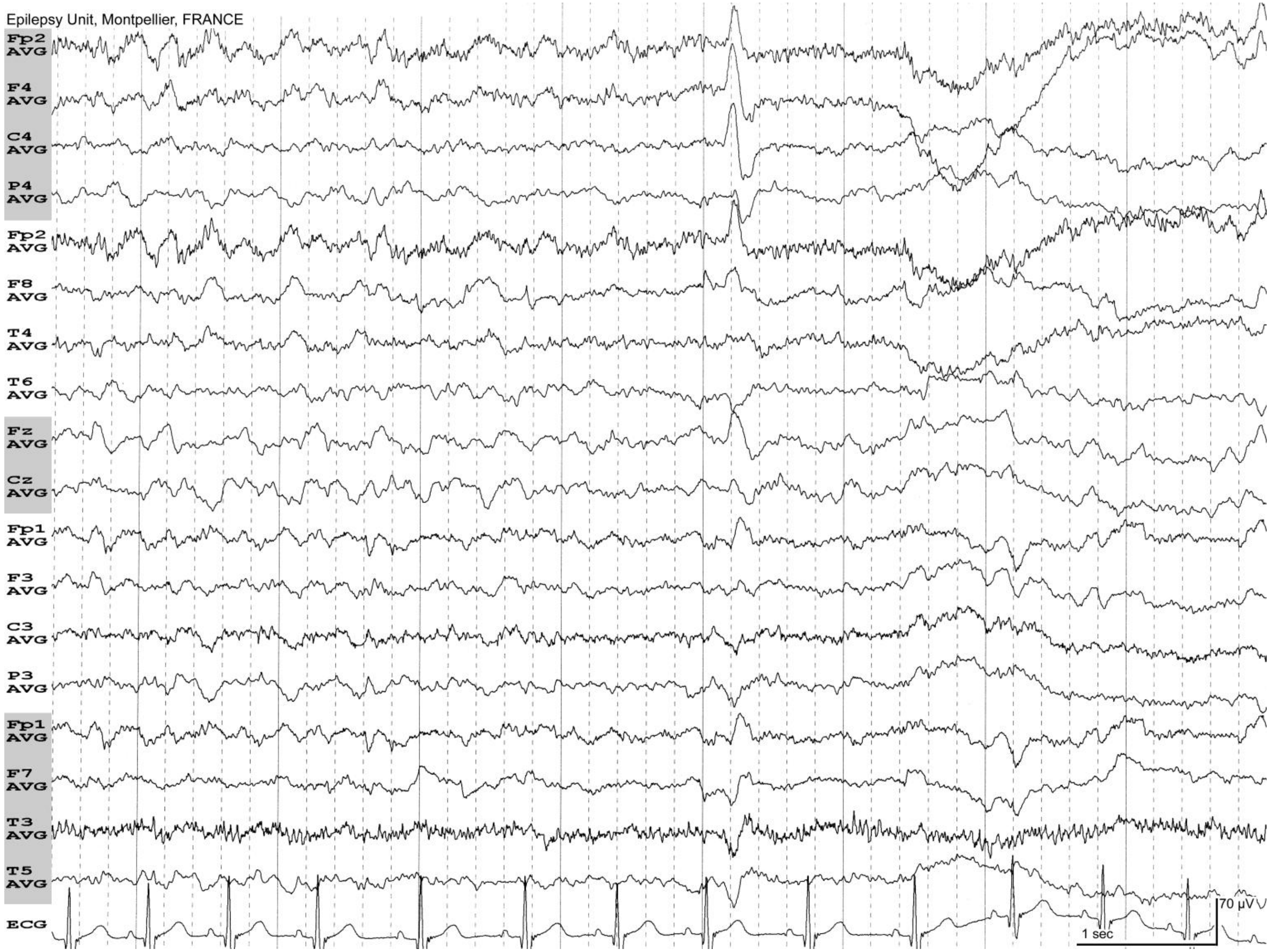

Average montage

EEG recorded at 17.5 years of age.

NREM sleep. Isolated biphasic complexes are observed on the fronto-central and vertex regions.

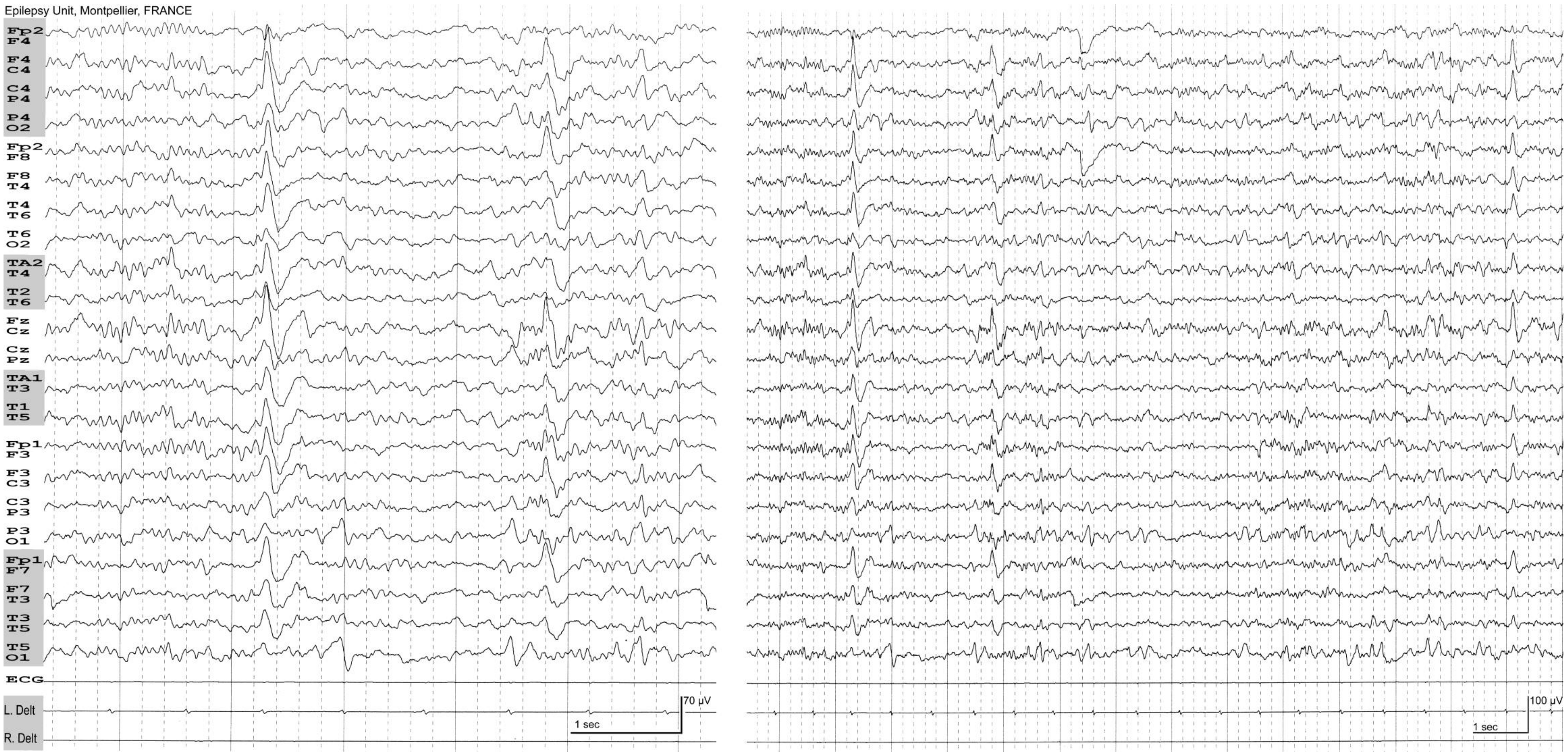

International 10–20 electrode placement system and supplementary anterior/inferior temporal electrodes

(TA1/TA2: Temporal-Anterior; T1/T2: zygomatic electrode)

EEG recorded at 17.5 years of age.

NREM sleep. A series of biphasic complexes is present. Sleep spindles are more prominent on the left hemisphere.

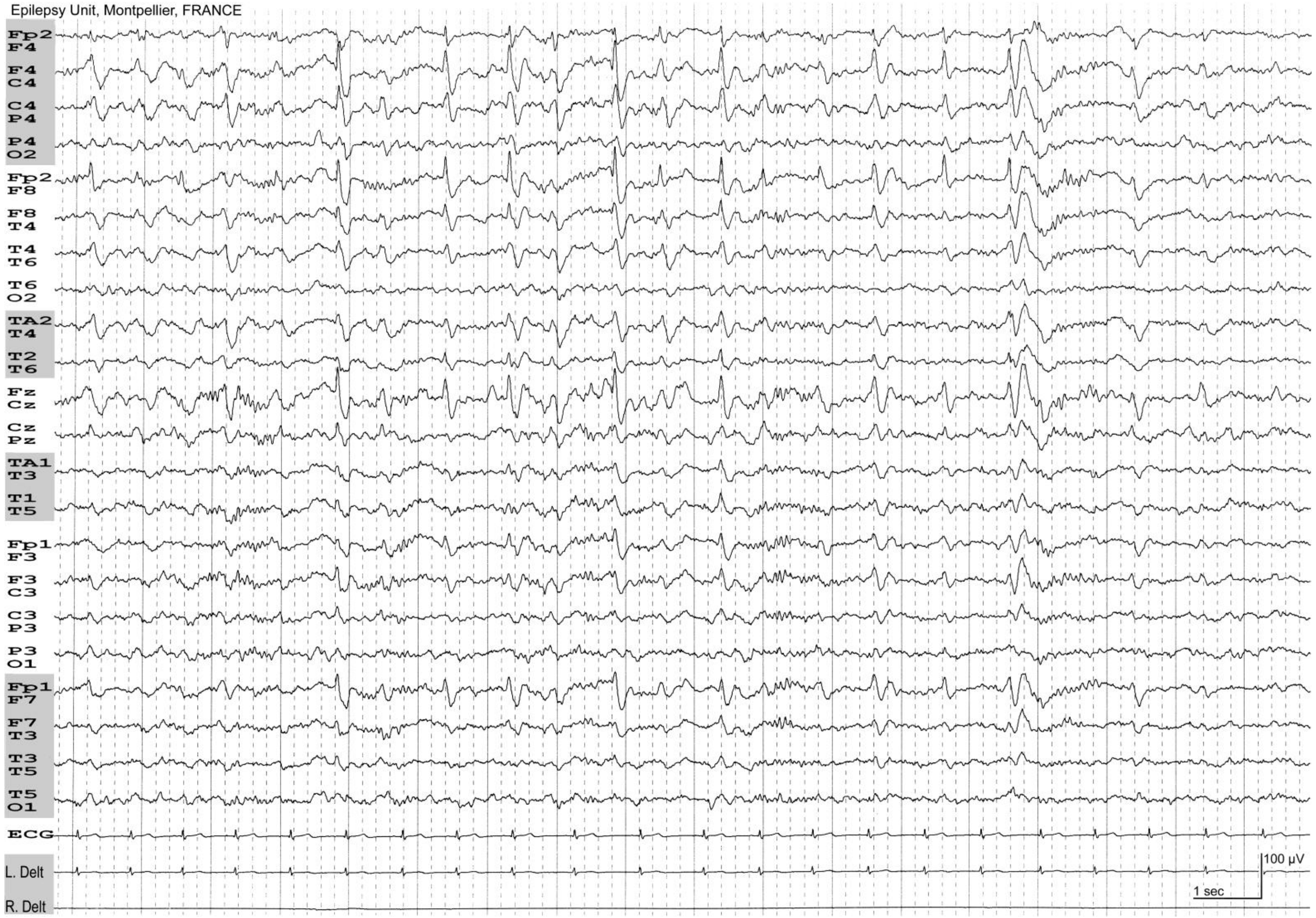

International 10–20 electrode placement system and supplementary anterior/inferior temporal electrodes (TA1/TA2: Temporal-Anterior; T1/T2: zygomatic electrode)
